# Impact of duck stocking density on growth performance, digestive enzymes, blood biochemistry, and antioxidant capacity of the *Labeo rohita reared in an* integrated ponds system

**DOI:** 10.1371/journal.pone.0294064

**Published:** 2023-11-08

**Authors:** Iqra Anwer, Muhammad Hafeez-ur-Rehman, Farzana Abbas, Shagufta Saeed

**Affiliations:** 1 Department of Fisheries and Aquaculture, University of Veterinary and Animal Sciences, Lahore, Pakistan; 2 Institute of Biochemistry and Biotechnology, University of Veterinary and Animal Sciences, Lahore, Pakistan; Benha University, EGYPT

## Abstract

This study investigated the effects of duck stocking density (SD) on growth performance, meat quality, hematology, serum metabolites, and antioxidant status of *L*. *rohita* reared in an integrated pond system. A total of 9000 fingerlings of *L*. *rohita* average weighing 150.5±1 grams were reared in nine ponds (n = 1000/pond) and randomly allotted one of three SDs under a completely randomized design. The stocking densities were 100 (SD-100), 150 (SD-150), and 200 (SD-200) ducks/pond and each group had three replicates. The final body weight, weight gain, and specific growth rates were greater (*p*<0.05) in SD-200 than in the rest of the treatments. The activities of proteases and amylase were also greater (*p*<0.05) for SD-200 than for the rest of the SD. However, hematological parameters like red blood cells, white blood cells, hematocrit, met hematocrit, and hemoglobin contents were not influenced (*p*>0.05) by SD in ponds. The serum total protein, albumin, globulin, creatinine, and glucose contents were also similar (*p*>0.05) across the ponds. However, blood urea nitrogen was higher (*p*<0.05) in SD-200 than in the rest of the SD. Moreover, alanine transaminase, aspartate transaminase, and alkaline phosphatase activities were linearly decreased (*p*<0.05) with increasing SD across the ponds. The changes in catalases and superoxide dismutase were non-significant (*p*>0.05) among the groups. However, glutathione peroxidase was greater for SD-200 than for the rest of the SD. The carcass compositional characteristics such as dry matter, protein, fat, and ash contents were not changed (*p*>0.05) by varying SD. In conclusion, SD over 200 birds/pond improved growth performance, digestive enzyme functions, and oxidative capacity without any negative impact on the hematology and carcass composition of *L*. *rohita*.

## Introduction

Aquaculture is rapidly emerging as an industry that plays a crucial role in ensuring global food security by providing a reliable supply of high-quality protein for human consumption [1]. Aquaculture production systems in developing countries such as Pakistan range from extensive polyculture farming systems to intensive commercial rearing practices [2, 3]. Intensive aquaculture has demonstrated the ability to achieve remarkable growth performance within a relatively short timeframe [3]. However, the sustainability of such production systems is being called into question because of increasing environmental challenges [4] and resource constraints such as higher feeding costs (70–80% of total production cost), availability of feed ingredients, and their quality [5]. Integrated aquaculture farming systems evolve fish rearing alongside other livestock or crop species. Such operations cut production costs by utilizing animal excreta nutrients to boost pond productivity and, ultimately, fish growth [6]. Efficient use of energy, water, land, and feed resources, as well as reduced commercial fertilizer inputs, could be key to long-term healthy aquatic food production [7]. Ecological intensification is proposed to achieve the long-term goals of food production systems by integrating anthropogenic inputs with ecosystem services [8]. Sustainable intensification is important in adapting to global climate change [9].

Fish blood parameters and stress levels are linked to growth regulation [10]. Stocking density has also been shown to influence blood hematology, a critical parameter in determining fish physiological performance [11, 12]. Furthermore, stress caused by stocking density affects fish physiological performance, influencing growth, survival, body proximate composition, and health in farm-raised catfish [11, 13]. The fish stocking density in a pond is well optimized in several studies with varying success [14–16]. To the best of the authors’ knowledge, no studies have assessed the influence of the ducks stocking density on the growth performance and physiological responses of *L*. *rohita* reared in an integrated pond system was limited. Moreover, pond integration with livestock species manure such as duck dropping is a common practice in the developing world, and information regarding stocking optimization is also scarce. *L*. *rohita*, is one of the major species that have been widely subjected to pond culturing in developing countries such as Pakistan because of good meat quality and higher consumer preference [17]. Rohu is typically subjected to higher stocking due to the larger feeding niche that extends from column to bottom [18]. Keeping all in view, the current study aimed to evaluate the influence of SD on growth performance, digestive enzyme activities, blood biochemistry, meat quality, and antioxidant capacity of the *L*. *rohita* reared in an integrated pond system.

## Materials and methods

### Ethical approval, experimental design, and husbandry practices

The current research was carried out at the aquaculture research station, C block Ravi Campus Pattoki, UVAS Lahore. A total of 9000 fingerlings of *L*. *rohita* average weighing 150.5±1 grams were reared in nine ponds (n = 1000/pond) and randomly allotted one of three SDs under a completely randomized design. The stocking densities were 100 (SD-100), 150 (SD-150), and 200 (SD-200) ducks/pond and each group had three replicates. The ponds were rectangular with dimensions 24 feet in length, and 9 feet in width of each pond. Ducks were housed and reared in a group form after randomization. Each pond was specified for respective ducks’ stock throughout the experimental duration (110 days). Stocks were discharged in the respective ponds at 0:900 h after the morning feeding and collected back to cages at 17:00 h of the day. The ducks were fed on a commercial feed throughout the experiment and daily feed was offered at 7:00 and 18:00 h of the day. The ponds were fenced with wire to ensure the separation of the groups in their respective ponds. The water of each pond was drained biweekly and refilled with fresh clean water. The physical parameters were measured every day between 9:00 and 10:00 hours of the day by using a multi-parameter probe (HI 9829 Hanna). The recorded water temperature, pH, dissolved oxygen, conductivity, and total dissolved oxygen levels ranged between 17–19°C, 8.5–8.7, 6.3–6.4 mg L^−1^, 1225–1230, and 686–688 mg L^−1^ respectively. Fish harvesting was performed by the net method at the end of the experiment.

### Growth performance

The body weights were measured before the start of the feeding trial, and biweekly fishes from each pond were randomly sampled (200 fish/pond) on the day of termination of the experiment to calculate the body weight changes. The following Eqs 1–2 were used to calculate the growth performance.


Bodyweightgain(g)=Finalweight(g)−Initialweight(g) (1)

Specificgrowthrate=[(Finalweight−Initialweight)/growthtrialduration]×100 (2)

### Sample collection and laboratory analysis

At the end of the experiment, twenty fish from each pond were selected and anesthetized by using a 150 mg/L concentration of tricane methanesulphate (MS-222) according to Mushtaq et al. [3]. Blood samples from ten fishes/ponds were collected in plain tuberculin syringes by puncturing the caudal vasculature, centrifugated immediately at 3000 x *g* for 15 minutes, and harvested serum was stored at -20°C for further analysis. Furthermore, the caudal vein was punctured using EDTA-coated tuberculin syringes, and collected blood samples were immediately analyzed for hematological parameters using an automated hematology analyzer (MEK6550). The blood glucose level was measured through commercial kits (21503, Biosystems, Barcelona, Spain). Five fishes from each pond were dissected for organ collection (intestine, kidney, liver, gills, and pancreas) and biological indices, and the rest five were homogenized in a meat mincer (ANEX, AG 3060) to evaluate the meat quality. The collected organs and meat samples were stored at -20°C in labeled plastic airtight zipper bags until further analysis. To calculate the dry matter contents, the meat samples were dried for 48 h at 55°C in a forced-air oven. Using a Foss grinder, dried meat samples were ground and passed through a 1 mm sieve (CT 293 Cyclotec, Denmark). The crude protein (Method 976.06) and fat contents of meat samples were determined using the AOAC, (1990) standard procedures (Soxtec procedure, Tecator, Hoganas, Sweden; method 920.29). To determine the ash content of ground meat and feed samples, they were ignited in a muffle furnace at 620°C for 3 hours.

### Antioxidant assay

The 2 g muscle and liver samples of each fish were homogenized with 6 ml phosphate buffer (pH 7.4), filtered (Whatman filter paper no. 1), and centrifuged at 10,000 g for 15 min. All the steps to measure the enzymatic activities were carried out at 4°C. The superoxide dismutase (SOD) activity to restrain nitroblue tetrazole was estimated according to the method described by Giannopolitis and Ries [19]. The activities of catalase (CAT) and glutathione peroxidase (GPx) were estimated by measuring the concentration of decomposed hydrogen peroxide at 240 nm and 470 nm respectively, as described by Mushtaq, Fatima [20]. The activities of ALT, AST, and ALP in serum samples were estimated by using commercial kits (AL1205, AS3804, AP9764; Randox Laboratories Ltd).

### Digestive enzyme activity

The collected intestinal samples were homogenized immediately after mixing with sodium phosphate buffer (0.05 M, pH 7.0; wt: vol = 1:9), centrifuged at 6000 ×*g* for 10 min at 4°C, and the supernatant was harvested to test the intestinal digestive enzymes activities. Amylase activity was determined using the 3,5-dinitro salicylic acid method as described by Thongprajukaew, Kovitvadhi [21]. Briefly, 0.5 mL of the supernatant was incubated with 1% starch solution (0.5 mL) at 37°C for 30 min. Approximately 1 mL of 3,5-dinitro salicylic acid reagent was added to the mixture, incubated in boiling water, soaked for 5 min, and cooled to ambient temperature. After the solution was diluted with an appropriate amount of double-distilled water, the absorbance was measured at a 540 nm spectrophotometer. One unit of amylase activity shows the presence of an enzyme that released 1 μg of reducing sugar per minute. The protease enzyme activity was estimated according to the method by Cupp-Enyard [22] by using bovine serum albumin as the substrate. The absorbance values were recorded at 680 nm.

### Statistical analysis

Data were analyzed using ANOVA techniques of SAS (Online version 2023) with stocking densities as a fixed factor/independent variable. Means were compared and separated by the Tukey test with a significant level of *p*<0.05.

## Results

### Growth performance

According to Table 1, the duck’s SD influenced (*p*<0.05) the fish survival rates and growth performance. The survival rates for the SD-200, SD-150, and SD-100 groups were recorded as 97.75%, 96.75%, and 95.25%, respectively. The growth parameters like final body weight, body weight gain, and specific growth rate were linearly increased (*p*<0.05) with increasing ducks SD in ponds.

**Table 1:** Growth performance of *L*.*rohita* reared in integrated ponds containing different duck stocking densities.

Parameters	Stocking Densities^1^			SEM^2^	p-linear
	SD-100	SD-150	SD-200		
Initial body weight (g)	150.43	150.51	150.86	0.368	0.532
Final body weight (g)	565.71^a^	608.50^b^	654.91^c^	0.582	0.000
Body weight gain (g)	415.27^a^	457.92^b^	504.01^c^	0.853	<0.0001
Weight gain (%)	73.41^a^	75.26^b^	76.96^c^	0.814	0.000
Specific growth rate (g)	3.77^a^	4.16^b^	4.58^c^	0.007	0.000
Survival rate (%)	95.25^a^	96.75^b^	97.75^c^	0.250	0.013

^1^Stocking densities = ponds containing stocking density of ducks @ 100 ducks/pond (SD-100), 150 ducks/pond (SD-150), and 200 ducks/pond (SD-200); and

^2^SEM = standard error of means.

### Digestive enzyme activities

The means along with pooled stander error of means of digestive enzyme activities of *L*. *rohita* reared under different SD of ducks are given in Table 2. The activities of proteases and amylase were significantly greater (*p*<0.05) for SD-200 than the rest of the SD of the ducks in ponds.

**Table 2:** Digestive enzyme activities of *L*.*rohita* reared in integrated ponds containing different duck stocking densities.

Parameters	Stocking Densities^1^			SEM^2^	p-linear
	SD-100	SD-150	SD-200		
Protease Activity (μg/min)	130.45^a^	143.8^b^	141.25^c^	0.745	0.002
Amylase activity (μg /min)	8.71^a^	10.73^b^	11.76^c^	0.437	0.016

^1^Stocking densities = ponds containing stocking density of ducks @ 100 ducks/pond (SD-100), 150 ducks/pond (SD-150), and 200 ducks/pond (SD-200); and

^2^SEM = standard error of means.

### Hematology and serum metabolites

The hematological analysis and serum metabolites of *L*. *rohita* reared under different ducks SD are given in Tables 3 & 4 respectively. The means of RBCs, WBCs, HCT, MCHC, and Hb were similar (*p*>0.05) among the groups. Similarly, serum total protein, albumin, globulin, creatinine, and glucose contents were also similar (*p*>0.05) across the groups. The blood urea nitrogen contents were greater (*p*<0.05) in the SD-200 group across the treatments. Moreover, ALT, AST, and ALP activities were linearly decreased (*p*<0.05) with increasing SD across the groups.

**Table 3:** Hematology of *L*.*rohita* reared in integrated ponds containing different duck stocking densities.

Parameters	Stocking Densities^1^			SEM^2^	p-linear
	SD-100	SD-150	SD-200		
WBC (106/μl)^3^	26.10	27.55	28.00	0.582	0.113
RBC (106/μl)^4^	2.83	2.95	2.94	0.032	0.130
Hb (g/dl)^5^	8.23	8.95	8.32	0.017	0.125
HCT (%)^6^	43.19	44.05	43.25	1.023	0.407
MCHC (g/dl)^7^	26.12	25.24	26.98	0.655	0.141

^1^Stocking densities = ponds containing stocking density of ducks @ 100 ducks/pond (SD-100), 150 ducks/pond (SD-150), and 200 ducks/pond (SD-200)

^2^SEM = standard error of means

^3^WBC = White blood cells

^4^RBC = Red blood cells

^5^Hb = Hemoglobin

^6^HCT = Hematocrit; and

^7^MCHC = Met hematocrit.

**Table 4:** Serum metabolites of *L*.*rohita* reared in integrated ponds containing different duck stocking densities.

Parameters	Stocking Densities^1^			SEM^2^	p-linear
	SD-100	SD-150	SD-200		
Total protein (g/dl)	5.53	5.83	6.02	0.235	0.551
Albumin (g/dl)	2.92	3.12	2.95	0.143	0.250
Globulin (g/dl)	3.21	3.42	3.34	0.537	0.228
Creatinine (g/dl)	3.32	3.31	3.41	0.042	0.390
Blood urea nitrogen (mg/dl)	4.21^a^	5.43^b^	5.97^c^	0.120	0.011
Glucose (mg/dl)	80.01	81.51	80.99	0.822	0.251
ALP (IU/ml)^3^	29.94^c^	27.24^b^	26.12^b^	0.962	0.021
ALT (IU/ml)^4^	50.68^c^	48.76^b^	46.75^b^	1.586	0.014
AST (IU/ml)^5^	97.25^c^	95.23^b^	93.57^b^	1.651	0.023

^1^Stocking densities = ponds containing stocking density of ducks @ 100 ducks/pond (SD-100), 150 ducks/pond (SD-150), and 200 ducks/pond (SD-200)

^2^SEM = standard error of means

^3^ALP = Alanine phosphatase

^4^ALT = Alanine transaminase; and

^5^AST = Aspartate transaminase.

### Oxidative stress-associated biomarkers

The results of the antioxidant enzymes of *L*. *rohita* are given in Table 5. The changes in catalases and SOD in both liver and muscle were non-significant (*p*>0.05) among the groups. However, means of glutathione peroxidase were influenced (*p*<0.05) by SD for both muscle and liver. The glutathione peroxidase was greater for SD-200 than for the rest of SD.

**Table 5:** Oxidative stress-associated biomarkers of *L*.*rohita* reared in integrated ponds containing different duck stocking densities.

Parameters	Stocking Densities^1^			SEM^2^	p-linear
	SD-100	SD-150	SD-200		
*Liver*					
Superoxide dismutase (μ/mg)	5.98	5.87	6.32	0.054	0.159
Catalases (μ/mg)	77.23	76.89	76.84	0.234	0.153
Glutathione peroxidase (μ/mg)	228.89^a^	236.02^b^	243.63^c^	2.865	0.026
*Muscle*					
Superoxide dismutase (μ/mg)	6.23	6.01	6.13	0.003	0.167
Catalases (μ/mg)	71.42	71.84	72.48	0.432	0.132
Glutathione peroxidase (μ/mg)	221.6^a^	228.03^b^	251.34^c^	1.593	0.017

^1^Stocking densities = ponds containing stocking density of ducks @ 100 ducks/pond (SD-100), 150 ducks/pond (SD-150), and 200 ducks/pond (SD-200); and

^2^SEM = standard error of means.

### Meat chemical composition

According to Table 6, the carcass compositional characteristics such as dry matter, protein, fat, and ash contents of fish were not changed (*p*>0.05) by varying SD of the ducks in ponds.

**Table 6:** Carcass composition of *L*.*rohita* reared in integrated ponds containing different duck stocking densities.

Parameters	Stocking Densities^1^			SEM^2^	p-linear
	SD-100	SD-150	SD-200		
Dry matter (%)	75.75	75.24	74.25	0.251	0.162
Crude protein (%)	16.43	16.52	16.51	0.027	0.081
Crude fat (%)	5.55	4.97	5.32	0.422	0.269
Ash (%)	4.32	5.27	5.55	0.193	0.235

^1^Stocking densities = ponds containing stocking density of ducks @ 100 ducks/pond (SD-100), 150 ducks/pond (SD-150), and 200 ducks/pond (SD-200)

^2^SEM = standard error of means.

## Discussion

To our knowledge, this is the first study describing responses of *L*. *rohita* to different SD in an integrated pond-rearing system and to show how SD could affect fish growth performance, carcass composition, and health parameters. In integrated aquaculture systems, the co-cultivation of ducks and fish in the same pond is performed. Ducks not only provide an additional source of income but also contribute to the nutrient dynamics of the pond [23]. Moreover, such farming practices are commonly practiced by smallholders, particularly in developing countries [24]. However, the stocking density of ducks can significantly affect the growth performance of fish. Therefore, the current experiment hypothesized that greater SD can improve the growth performance of *L*. *rohita* by increasing pond productivity. This discussion aims to explore the impact of duck stocking density in ponds on fish performance and health. As expected, high SD (SD-200) improved body weight gain and specific growth rates in the current experiment. These results can be associated with a greater number of ducks in ponds serving as a source of organic matter and nutrients through their droppings, which contain protein, phosphorus, and other essential elements. Adequate nutrient availability can enhance primary productivity, leading to an abundant food source for fish. Several studies reported that higher dietary protein intake improved the growth performance of the fish [25–27]. It is well established that higher availability of the protein enhances the performance of the fish [28]. The improved digestive enzyme activities of the current study are augmenting the finding regarding growth performance as these are established indicators of nutrient digestibility, utilization, and assimilation in different fish species [29, 30]. Greater nutrient availability is presumably due to the enrichment of the pond water with floating nutrients from ducks’ droppings resulting in better growth performance with better digestion indicators [29, 31]. The increased protease and amylase activities for the SD-200 group in our experiment, support the above points. Thus, we can infer that increasing SD resulting more nutrient availability to improve the growth performance of the *L*. *rohita*. Blood metabolites serve as biomarkers to investigate health and disease conditions. Several factors including fluctuations in environments factors cause changes in the normal physiological levels of these metabolites [32]. Moreover, it is well established that the digested nutrients and metabolic products are directly absorbed in the blood through portal circulation and changed the blood’s biochemical properties [3]. Although the means of RBCs, WBCs, HCT, MCHC, and Hb were similar across different groups in the current experiment. The results also showed that levels of the serum metabolites such as serum total protein, albumin, globulin, creatinine, and glucose were also not changed across the groups. However, blood urea nitrogen was greater in SD-200 than in the rest of the groups. In the current research, we hypothesized that SD might affect the blood cells and serum metabolites of *L*. *rohita*. As results show that SD had no effects on hematological items and blood metabolites (except blood urea nitrogen) of growing fish. In addition, all of the measured parameters were in the normal range for *L*. *rohita* [33]. There are no data regarding the impacts of SD on blood hematology and metabolites of fish. Blood glucose is well-known stress-associated biomarker in fishes [34], and serum total protein has a connection with nutrient metabolism, and the immune system of fish [35]. The concentration of WBCs reflects the lysozyme activities and health status of the fish [20]. In the current experiment, increasing SD linearly decreased ALT, AST, and ALP activities suggesting healthy fish with better liver function and antioxidant capacity [3].

Oxidative stress is a pathophysiological condition that triggers irreversible tissue damage by reactive oxygen species [36]. The fish redox system basing upon certain enzymes such as SOD, CAT, and GPx which play a scavenging role to detoxify the reactive oxygen species [3]. In our experiment, the activities of SOD, and CAT for both muscle and liver were not changed across the groups. However, the level of GPx was significantly greater in SD-200 than in the rest of the groups suggesting that increasing SD produced stronger *L*. *rohita*. Studies in different fish species reported improved GPx activities meaning stronger fish with better antioxidant status [37, 38]. Several studies documented stocking density affects the antioxidative status of aquatic animals [39, 40]. The proximate analysis showed that fish meat protein, dry matter, fat, and ash contents were similar across the groups in the current experiment suggesting that the muscle composition of *L*. *rohita* has no sensitivity to SD.

## Conclusion

Based on the results, the higher stocking density of the ducks in an integrated pond improved the growth performance, and digestive enzyme functions of *L*. *rohita*. In addition, strengthens the oxidative burst by increasing the concentrations of GPX which is a key enzyme to activate the redox defense system. The liver health indicators (ALT, AST, and ALP) were improved by increasing the ducks stocking density in the pond. However, the hematological parameters and carcass chemical composition of the fish were not influenced by varying duck stocking densities. Further studies are warranted to explore the higher duck stocking density and their influence on the growth performance of different carbs in an integrated pond-rearing setup.

## References

[1] Kurniawan SB, Abdullah SRS, Imron MF, Ahmad A, Mohd Said NS, Mohd Rahim NF, et al. Potential of valuable materials recovery from aquaculture wastewater: an introduction to resource reclamation. Aquaculture Research. 2021;52(7):2954–62.

[2] Rehman A, Deyuan Z, Hena S, Chandio AA. Do fisheries and aquaculture production have dominant roles within the economic growth of Pakistan? A long-run and short-run investigation. British Food Journal. 2019;121(8):1926–35.

[3] Mushtaq M, Fatima M, Shah SZH, Khan N, Naveed S, Khan M. Evaluation of dietary selenium methionine levels and their effects on growth performance, antioxidant status, and meat quality of intensively reared juvenile Hypophthalmichthys molitrix. Plos one. 2022;17(9):e0274734. doi: 10.1371/journal.pone.0274734 36112655 PMC9480980

[4] Boyd CE D’Abramo LR, Glencross BD, Huyben DC, Juarez LM, Lockwood GS, et al. Achieving sustainable aquaculture: Historical and current perspectives and future needs and challenges. Journal of the World Aquaculture Society. 2020;51(3):578–633.

[5] Albrektsen S, Kortet R, Skov PV, Ytteborg E, Gitlesen S, Kleinegris D, et al. Future feed resources in sustainable salmonid production: A review. Reviews in aquaculture. 2022;14(4):1790–812.

[6] Thomas M, Pasquet A, Aubin J, Nahon S, Lecocq T. When more is more: taking advantage of species diversity to move towards sustainable aquaculture. Biological reviews. 2021;96(2):767–84. doi: 10.1111/brv.12677 33320418

[7] Xu C, Lv Z, Shen Y, Liu D, Fu Y, Zhou L, et al. Metagenomic insights into differences in environmental resistome profiles between integrated and monoculture aquaculture farms in China. Environment International. 2020;144:106005. doi: 10.1016/j.envint.2020.106005 32739516

[8] Stenton-Dozey JM, Heath P, Ren JS, Zamora LN. New Zealand aquaculture industry: research, opportunities and constraints for integrative multitrophic farming. New Zealand Journal of Marine and Freshwater Research. 2021;55(2):265–85.

[9] Godfray HCJ. The debate over sustainable intensification. Food Security. 2015;7:199–208.

[10] Sheng Y, Hua ZY, Yang Z, Wei XL, Sheng YJ, Jia HL, et al. Effects of acute hypoxic stress on biochemical parameters, immune regulation and metabolic capacity of the blood in genetically improved farmed tilapia (GIFT, Oreochromis niloticus). Journal of Applied Ichthyology. 2019;35(4):978–86.

[11] Refaey MM, Li D, Tian X, Zhang Z, Zhang X, Li L, et al. High stocking density alters growth performance, blood biochemistry, intestinal histology, and muscle quality of channel catfish Ictalurus punctatus. Aquaculture. 2018;492:73–81.

[12] Fauji H, Budiardi T, Ekasari J. Growth performance and robustness of African Catfish Clarias gariepinus (Burchell) in biofloc‐based nursery production with different stocking densities. Aquaculture Research. 2018;49(3):1339–46.

[13] Battisti EK, Rabaioli A, Uczay J, Sutili FJ, Lazzari R. Effect of stocking density on growth, hematological and biochemical parameters and antioxidant status of silver catfish (Rhamdia quelen) cultured in a biofloc system. Aquaculture. 2020;524:735213.

[14] Toko I, Fiogbe ED, Koukpode B, Kestemont P. Rearing of African catfish (Clarias gariepinus) and vundu catfish (Heterobranchus longifilis) in traditional fish ponds (whedos): Effect of stocking density on growth, production and body composition. Aquaculture. 2007;262(1):65–72.

[15] Oké V, Goosen NJ. The effect of stocking density on profitability of African catfish (Clarias gariepinus) culture in extensive pond systems. Aquaculture. 2019;507:385–92.

[16] Lu J, Li S, He X, Tang R, Li D. An in-pond tank culture system for high-intensive fish production: Effect of stocking density on growth of grass carp (Ctenopharyngodon idella Valenciennes, 1844) and blunt snout bream (Megalobrama amblycephala Yih, 1955). Aquaculture. 2022;549:737808.

[17] Chaudhary A, Ahmad Q-u-A, Akram AM, Iqtedar M, Qazi JI. Effect of Sphingomonas sp., as a Probiotic on Survival, Growth and Biochemical Constituents of Vibrio anguillarum Challenged Labeo rohita Fingerlings. Pakistan Journal of Zoology. 2021;53(6).

[18] Hossain MB, Nur A-AU, Ahmed MM, Ullah MA, Albeshr MF, Arai T. Growth, Yield and Profitability of Major Carps Culture in Coastal Homestead Ponds Stocked with Wild and Hatchery Fish Seed. Agriculture. 2022;12(8):1131.

[19] Giannopolitis CN, Ries SK. Superoxide dismutases: II. Purification and quantitative relationship with water-soluble protein in seedlings. Plant physiology. 1977;59(2):315–8. doi: 10.1104/pp.59.2.315 16659840 PMC542388

[20] Mushtaq M, Fatima M, Shah SZH, Khan N, Naveed S, Khan M. Effects of sodium selenite, selenium methionine, and selenium yeast on growth performance, carcass composition, blood biochemistry, and antioxidant status of intensively reared Hypophthalmichthys molitrix. Aquaculture Reports. 2022;24:101182. 10.1371/journal.pone.0274734 PMC9480980 36112655

[21] Thongprajukaew K, Kovitvadhi U, Kovitvadhi S, Somsueb P, Rungruangsak-Torrissen K. Effects of different modified diets on growth, digestive enzyme activities and muscle compositions in juvenile Siamese fighting fish (Betta splendens Regan, 1910). Aquaculture. 2011;322:1–9.

[22] Cupp-Enyard C. Sigma’s non-specific protease activity assay-casein as a substrate. JoVE (Journal of Visualized Experiments). 2008(19):e899. doi: 10.3791/899 19066538 PMC2872977

[23] Ozretich RW, Wood CL, Allan F, Koumi AR, Norman R, Brierley AS, et al. The potential for aquaculture to reduce poverty and control schistosomiasis in Côte d’Ivoire (Ivory Coast) during an era of climate change: a systematic review. Reviews in Fisheries Science & Aquaculture. 2022;30(4):467–97.

[24] Skillicorn P, Spira W, Journey W. A new aquatic farming system for developing countries. The world bank group. 1993;76:76.

[25] Yang S-D, Liou C-H, Liu F-G. Effects of dietary protein level on growth performance, carcass composition and ammonia excretion in juvenile silver perch (Bidyanus bidyanus). Aquaculture. 2002;213(1–4):363–72.

[26] Yang SD, Lin TS, Liou CH, Peng HK. Influence of dietary protein levels on growth performance, carcass composition and liver lipid classes of juvenile Spinibarbus hollandi (Oshima). Aquaculture Research. 2003;34(8):661–6.

[27] Weththasinghe P, Hansen J, Nøkland D, Lagos L, Rawski M, Øverland M. Full-fat black soldier fly larvae (Hermetia illucens) meal and paste in extruded diets for Atlantic salmon (Salmo salar): Effect on physical pellet quality, nutrient digestibility, nutrient utilization and growth performances. Aquaculture. 2021;530:735785.

[28] Yu H, Liang H, Ren M, Ge X, Ji K, Huang D, et al. A study to explore the effects of low dietary protein levels on the growth performance and nutritional metabolism of grass carp (Ctenopharyngodon idella) fry. Aquaculture. 2022;546:737324.

[29] Kari ZA, Kabir MA, Dawood MA, Razab MKAA, Ariff NSNA, Sarkar T, et al. Effect of fish meal substitution with fermented soy pulp on growth performance, digestive enzyme, amino acid profile, and immune-related gene expression of African catfish (Clarias gariepinus). Aquaculture. 2022;546:737418.

[30] Ezhilmathi S, Ahilan B, Uma A, Felix N, Cheryl A, Somu Sunder Lingam R. Effect of stocking density on growth performance, digestive enzyme activity, body composition and gene expression of Asian seabass reared in recirculating aquaculture system. Aquaculture Research. 2022;53(5):1963–72.

[31] Zhang Y, Guan C, Li Z, Luo J, Ren B, Chen C, et al. Review of Rice–Fish–Duck Symbiosis System in China—One of the Globally Important Ingenious Agricultural Heritage Systems (GIAHS). Sustainability. 2023;15(3):1910.

[32] Khan M, Rashid MA, Yousaf MS, Naveed S, Mohsin I, Rehman HU, et al. Effects of physical forms of a high grain-based diet on fattening performance, ruminal health, feeding behaviour, nutrient digestibility and carcass traits of finishing Lohi lambs. Archives of Animal Nutrition. 2023:1–16. doi: 10.1080/1745039X.2023.2179296 36880568

[33] Alaguprathana M, Poonkothai M. Haematological, biochemical, enzymological and histological responses of Labeo rohita exposed to methyl orange dye solution treated with Oedogonium subplagiostomum AP1. Environmental Science and Pollution Research. 2021;28:17602–12. doi: 10.1007/s11356-020-12208-7 33400116

[34] Long L, Zhang H, Ni Q, Liu H, Wu F, Wang X. Effects of stocking density on growth, stress, and immune responses of juvenile Chinese sturgeon (Acipenser sinensis) in a recirculating aquaculture system. Comparative Biochemistry and Physiology Part C: Toxicology & Pharmacology. 2019;219:25–34. 30738212 10.1016/j.cbpc.2019.02.002

[35] Tahmasebi-Kohyani A, Keyvanshokooh S, Nematollahi A, Mahmoudi N, Pasha-Zanoosi H. Effects of dietary nucleotides supplementation on rainbow trout (Oncorhynchus mykiss) performance and acute stress response. Fish Physiology and Biochemistry. 2012;38:431–40. doi: 10.1007/s10695-011-9524-x 21671024

[36] Bhattacharyya A, Chattopadhyay R, Mitra S, Crowe SE. Oxidative stress: an essential factor in the pathogenesis of gastrointestinal mucosal diseases. Physiological reviews. 2014;94(2):329–54. doi: 10.1152/physrev.00040.2012 24692350 PMC4044300

[37] Siddik MA, Vatsos IN, Rahman MA, Pham HD. Selenium-Enriched Spirulina (SeE-SP) Enhance Antioxidant Response, Immunity, and Disease Resistance in Juvenile Asian Seabass, Lates calcarifer. Antioxidants. 2022;11(8):1572. doi: 10.3390/antiox11081572 36009291 PMC9404762

[38] Ibrahim MS, El‐gendy GM, Ahmed AI, Elharoun ER, Hassaan MS. Nanoselenium versus bulk selenium as a dietary supplement: Effects on growth, feed efficiency, intestinal histology, haemato‐biochemical and oxidative stress biomarkers in Nile tilapia (Oreochromis niloticus Linnaeus, 1758) fingerlings. Aquaculture Research. 2021;52(11):5642–55.

[39] Liu B, Jia R, Han C, Huang B, Lei J-L. Effects of stocking density on antioxidant status, metabolism and immune response in juvenile turbot (Scophthalmus maximus). Comparative Biochemistry and Physiology Part C: Toxicology & Pharmacology. 2016;190:1–8. doi: 10.1016/j.cbpc.2016.07.007 27497046

[40] Liu G, Zhu S, Liu D, Guo X, Ye Z. Effects of stocking density of the white shrimp Litopenaeus vannamei (Boone) on immunities, antioxidant status, and resistance against Vibrio harveyi in a biofloc system. Fish & Shellfish Immunology. 2017;67:19–26. 28522419 10.1016/j.fsi.2017.05.038

