# Correction: Impact of duck stocking density on growth performance, digestive enzymes, blood biochemistry, and antioxidant capacity of the *Labeo rohita reared in an* integrated ponds system

**DOI:** 10.1371/journal.pone.0317467

**Published:** 2025-01-08

**Authors:** Iqra Anwer, Muhammad Hafeez-ur-Rehman, Farzana Abbas, Shagufta Saeed

The first author, Iqra Anwer, is incorrectly noted as the corresponding author. The correct corresponding author is Muhammad Hafeez-ur-Rehman. Muhammad Hafeez-ur-Rehman’s email address is: mhafeezurehman@uvas.edu.pk.
